# Caloric Restriction and Health, Aging and Later-Life Outcomes in Older Adults

**DOI:** 10.1093/geroni/igaf122.1017

**Published:** 2025-12-31

**Authors:** Denise Houston, Jason Fanning, Stephen Kritchevsky

## Abstract

The prevalence of obesity (BMI ≥30 kg/m2) among older adults (65+ years) in the U.S. has almost doubled over the last three decades, with approximately 40% of older adults living with obesity. Lifestyle modifications of moderate caloric restriction (CR) have been shown to improve the metabolic and functional health in older adults (e.g., lowering blood pressure, fasting glucose, and interleukin-6, and improving cardiorespiratory fitness and gait speed) in the short-term. However, the long-term benefits and potential risks (e.g., loss of muscle and bone resulting in increased risk of sarcopenia and fractures) of CR have not been studied in older adults. This symposium will describe the outputs of an NIA-funded 3-year planning grant for use in the design of a 5-year CR trial in older adults with overweight or obesity. Fanning and colleagues will describe the feasibility and acceptability of the pilot intervention and associated self-monitoring technologies, as well as participant willingness to engage in a 5-year CR trial in older adults. Houston and colleagues will describe the metabolic adaptations in energy expenditure that occur in response to moderate CR in older adults. Hsu and colleagues will describe the effects of CR on a consensus derived panel of blood-based geroscience biomarkers that could be used as an intermediate endpoint in clinical trials of CR. Miller and colleagues will describe the development of an obesity- and aging-related health conditions index that captures the potential health effects of long-term studies of CR. Obesity and Aging Interest Group Sponsored Symposium

